# Sleep in Habitual Adult Video Gamers: A Systematic Review

**DOI:** 10.3389/fnins.2021.781351

**Published:** 2021-12-13

**Authors:** Chadley Kemp, Paula R. Pienaar, Dominique T. Rosslee, Gosia Lipinska, Laura C. Roden, Dale E. Rae

**Affiliations:** ^1^Health Through Physical Activity, Lifestyle and Sport Research Centre & Division of Physiological Sciences, Department of Human Biology, Faculty of Health Sciences, University of Cape Town, Cape Town, South Africa; ^2^Department of Public and Occupational Health, Amsterdam UMC, Amsterdam Public Health Research Institute, Vrije Universiteit Amsterdam, Amsterdam, Netherlands; ^3^Clinical Neuropsychology and Sleep Sciences, Department of Psychology, Faculty of Humanities, University of Cape Town, Cape Town, South Africa; ^4^Centre for Sport, Exercise and Life Sciences, Faculty of Health and Life Sciences, School of Life Sciences, Coventry University, Coventry, United Kingdom

**Keywords:** sleep patterns, sleep quality, insomnia, video games, electronic sports, cybergames

## Abstract

Video gaming is a popular, globally recognized phenomenon, played recreationally or competitively as esports. Gaming is a typically sedentary nighttime activity; therefore, the potential to impact sleep and health is high. Furthermore, there are limited studies on adult gamers, who represent the majority demographic in esports. This review examines evidence describing sleep in habitual adult gamers to understand the associated risk for cardiometabolic disease or the benefits to gaming performance. Three electronic databases (PubMed, Scopus, ISI Web of Science) were searched for peer-reviewed articles published between January 2000 – April 2020. Twelve studies reporting on sleep in habitual adult gamers were included. A narrative synthesis was employed to report results, owing to high levels of heterogeneity across the included studies. Gamers with higher gaming addiction scores were more likely to have shorter, poorer quality sleep and greater daytime sleepiness and insomnia scores than gamers with lower gaming addiction scores and non-gamers. In addition, high-volume gamers were more likely to have worsened sleep quantity and quality, with delayed sleep timing and increased prevalence of insomnia. Despite limitations in the design of the included studies, excessive gaming is broadly associated with worsened sleep parameters. Noteworthy is the lack of studies investigating cardiometabolic health in gamers. Future work should explore the relative contribution and associated risk that various games, genres, and timing of gaming activities have on sleep, physical and mental health, particularly in vulnerable gaming cohorts engaged with contemporary forms of gaming and esports.

## Introduction

Video gaming (henceforth, “gaming”) has become an increasingly popular and globally recognized phenomenon in recent years. Contemporary forms of gaming not only span multiple genres of games but multiple modalities, including computer, console, and mobile platforms (Newzoo, 2020). While gaming takes place both recreationally and competitively, most notable is the emergence of esports, which describes the conglomeration of competitive gaming titles and organized professional gaming events (McCutcheon et al., 2017). It is estimated that esports will have a total audience of 646 million people and global revenue of $1.6 billion by the end of 2023 (Newzoo, 2020). There are also schools of thought dedicated to extending health and performance research and optimizing the performance of esports athletes (Bonnar et al., 2019; Kemp et al., 2020).

In the past, gaming research has explored several domains, including the effect that games have on the psychological state (e.g., aggression), their use in cognitive training (e.g., gamification), and gaming addiction (Palaus et al., 2017; Pallavicini et al., 2018). However, most of these studies have investigated children and adolescents, in addition to the large body of literature investigating video game-based interventions in older adults. Despite reports indicating that most general gamers are now in their early thirties (Entertainment Software Association, 2019) and that most esports athletes are aged between 21 and 25 years (ESPN, 2017), few studies have considered young adults (i.e., 18 to 35 years). In addition, while there is less disparity in the sex distribution of gamers today, with females comprising 46% of the general gaming population (Entertainment Software Association, 2019), much of the contemporary research in gaming has been conducted among males. Thus, there is a need for research to consider young adults and females in the context of gaming.

It is stereotypical that gamers may spend many hours gaming at night, usually at the expense of sleep. This stereotype is corroborated by reports of some gamers spending up to 14 h playing games and achieving as little as 4 h of sleep in 24 h (Holden et al., 2018). These reports have prompted growing awareness and concern regarding the adverse health risks associated with excessive gaming (Holden et al., 2018). In particular, gaming is associated with longer sleep onset latency, short sleep duration, and poor sleep quality (Kristensen et al., 2021). There is also evidence describing altered sleep architecture from gaming, including reduced slow-wave sleep and rapid eye movement sleep (Higuchi et al., 2005; Dworak et al., 2007; Weaver et al., 2010). These pervasive effects on sleep are thought to be due to excessive screen exposure and attributed to the sleep-suppressing effect of catecholamines, which operate as part of the physiological arousal response to gaming (Weaver et al., 2010; Lam, 2014; Hale and Guan, 2015; Kim et al., 2016). However, there is little evidence of the mechanism that causes or underly these effects. Sleep disturbances may also manifest as sleep disorders, such as insomnias, and challenge the initiation and maintenance of healthy sleep. This could provoke problematic gaming behaviors, whereby gamers displace sleep with gaming activities as a way of coping with their sleeplessness (Kristensen et al., 2021). A vicious cycle may thus ensue, in which unhealthy sleep practices and problematic sleep perpetuate problematic gaming behaviors (Kristensen et al., 2021), translating to profound acute and chronic implications on cardiometabolic health. A recent study involving adolescent gamers found that curtailed sleep mediated the negative association identified between gaming addiction and abdominal obesity, which in turn was linked to higher blood pressure, altered lipid profiles, and insulin resistance (Turel et al., 2016). Chronic short and long sleep durations have been independently linked with greater all-cause mortality rates and risk for cardiometabolic disease (Yin et al., 2017). However, the implications around chronic gaming exposure as a contributor to adverse cardiometabolic health through poor sleep are not yet fully understood and are arguably multifaceted.

Another concern is the effect of gaming on circadian rhythms, including the sleep-wake cycle (Ceranoglu, 2014). Gamers may inadvertently phase delay their circadian rhythms in two ways: exposure to excessive light at night or gaming-induced behavior of late or delayed bedtimes. Short wavelength (i.e., blue) and bright light emitted from device screens may delay the onset of nocturnal melatonin secretion by the pineal gland and increase neurophysiologic arousal; this, in turn, delays sleepiness and thus, the onset of sleep, as well as the phase of other circadian-regulated processes (Gooley et al., 2011; Shechter et al., 2018). Through gaming at night, natural bedtimes might be delayed, which in turn may contribute to circadian disruption through a phenomenon known as social jetlag; a discrepancy between biological and social clocks, usually observed as the difference between sleep timing across working and non-working days (Wittmann et al., 2006; Chakradeo et al., 2018). Circadian disruption, regardless of the mechanism (i.e., shift work, jet lag, social jetlag), has been associated with increased risk for obesity, inflammation, insulin resistance, hypertension, type 2 diabetes mellitus, and cardiovascular disease (Lucassen et al., 2012; Wong et al., 2015; Parkar et al., 2019). Thus, gamers are likely to be at risk for short sleep, disrupted sleep, and disrupted circadian rhythms, which justifies the importance of studying cardiometabolic health in this population. Although chronic diseases may be more prevalent in older populations, the implicit risk associated with gaming behavior is profound, such that health risk factors presenting as an acute disease phenotype in young adults may translate to worsened chronic health implications later in life.

In addition to these cardiometabolic pathologies, there is also the issue regarding psychiatric disorders, which have historically been linked to light, circadian disruption, and impaired sleep (Wulff et al., 2010; Baron and Reid, 2014; Walker et al., 2020). In fact, disrupted sleep is a diagnostic criterion for most psychiatric disorders, including depression, bipolar disorder, anxiety, and post-traumatic stress disorder (American Psychiatric Association, 2013). More recently, however, circadian disruption and problematic sleep have also been linked to behavioral addictions, such as Internet Gaming Disorder (IGD), in which sleep problems are also thought to be mediators (Lam, 2014; Starcevic and Khazaal, 2017). A longitudinal study on a large sample of Singaporean children revealed that high gaming volume was one of several risk factors linked to gaming addiction (Gentile et al., 2011). The study also demonstrated that most (84%) pathological gamers remained addicted to gaming after 2-years and presented with several comorbid mood disorders, including depression, anxiety, and social phobias (Gentile et al., 2011). Gaming addictions have also been associated with poor sleep quality and insomnia, suggesting a bidirectional relationship between problematic gaming with mood disorders, circadian rhythms, and sleep problems (Lam, 2014). Therefore, problematic gaming can exacerbate affective symptoms in vulnerable individuals. Since parents may exercise their parental discretion regarding their child’s gaming activities, adults unable to self-regulate their gaming behaviors are arguably at greater risk for these more immediate pervasive effects.

There is also an abundant body of work describing positive aspects of gaming, including studies demonstrating cognitive benefits attributed to gaming (Buelow et al., 2015; Pallavicini et al., 2018). Cognitive processes critical to gaming may include elementary processes such as alertness, psychomotor and cognitive speed, and vigilance, in addition to executive processes, such as planning, problem-solving, working memory, and decision making (Gross and Grossman, 2010). While these processes may be susceptible to the effects of sleep restriction (Killgore, 2010; Lo et al., 2016), the degree to which adequate quantity and quality of sleep may impact gaming performance or limit the cognitive benefits attributed to gaming is unknown. For example, there is some evidence that sleep potentiates problem-solving in individuals challenged with a game involving logical reasoning (Beijamini et al., 2014). However, the decrement to executive processes following sleep restriction has been widely challenged, with the basis for the argument being that sleep loss affects the frontal lobes (the location of executive processing) more than most other regions of the brain (Tucker et al., 2010). Ultimately, managing sleep to preserve and optimize performance would presumably be an appealing motivation for gamers to reconsider their sleep-wake behavior and consequently act to negate deleterious long-term metabolic consequences resulting from excessive gaming.

Despite the plethora of studies involving gaming, there is a paucity of evidence involving habitual adult gamers. This cohort of predominantly young adults consists of individuals who self-identify as gamers or engage with gaming regularly and consistently for recreational (e.g., social gamers) or competitive (e.g., esports players) purposes. There is also a lack of research describing these individuals’ gaming behaviors, health status, and sleep patterns. The present systematic review aimed to examine the evidence describing sleep in habitual adult gamers, with a view of understanding what is known regarding the associated risk for cardiometabolic disease or the benefits concerning gaming performance or markers thereof.

## Methods

### Search Strategy

The search strategy used in the review process followed standardized procedures outlined in the Preferred Reporting Item for Systematic Review and Meta-Analysis (PRISMA) Statement (Liberati et al., 2009). Systematic literature searches were conducted across three electronic databases: PubMed, Scopus, and ISI Web of Science. These databases were used to retrieve studies published between 1 January, 2000 and 22 April, 2020. In addition, all citation libraries in the Web of Science Core Collection were considered, including Science Citation Index Expanded (SCI-EXPANDED), Social Sciences Citation Index (SSCI), Arts and Humanities Citation Index (A&HCI), and Emerging Sources Citation Index (ESCI) library indexes.

The electronic search strategy used the following terms: “esport*” or “e-sport*” or “electronic sport*” or “computer gam*” or “video gam*” or “internet gam*” or “gamer*” or “gaming” or “cybergam*” or “cyber gam*” and “sleep*” or “insomnia” in the title and abstract, or MeSH fields (“sleep” and “insomnia” only) for PubMed. Title, abstract, and keyword fields were used for Scopus, and topic fields were used for ISI Web of Science. The following search filters were applied to their respective database searches: Language: English; Publication Dates: 2000 – 2020; Species: Human; Document Type: Article; Source Type: Journal. The complete search strategy for each database is presented in Table 1.

**TABLE 1:** Systematic search strategy used in electronic databases.

Database	Search query
PubMed	esport*[tiab] OR e-sport*[tiab] OR “electronic sport*”[tiab] OR “computer gam*”[tiab] OR “video gam*”[tiab] OR “internet gam*”[tiab] OR gamer*[tiab] OR gaming[tiab] OR cybergam*[tiab] OR “cyber gam*”[tiab] AND sleep*[tiab] OR insomnia[tiab] OR sleep*[MeSH] OR insomnia[MeSH] Filters: Language: English; Species: Humans; Publication Dates: From 2000 to 2020
Scopus	TITLE-ABS-KEY (esport* OR e-sport* OR “electronic sport*” OR “computer gam*” OR “video gam*” OR “internet gam*” OR gamer* OR gaming OR cybergam* OR “cyber gam*”) AND TITLE-ABS-KEY (sleep* OR insomnia) AND PUBYEAR > 1999 AND PUBYEAR < 2021 AND (LIMIT-TO (SRCTYPE, “j”)) AND (LIMIT-TO (DOCTYPE, “ar”)) AND (LIMIT-TO (LANGUAGE, “English”))
ISI Web of Science	TS = (esport* OR e-sport* OR “electronic sport*” OR “computer gam*” OR “video gam*” OR “internet gam*” OR gamer* OR gaming OR cybergam* OR “cyber gam*”) AND TS = (sleep* OR insomnia) Limits: Language: English; Timespan 2000 – 2020; Document types: Article

### Eligibility Criteria

Original peer-reviewed research studies examining or reporting on sleep in habitual adult gamers were considered for inclusion in the present review. Habitual gamers were defined as individuals who self-identified as gamers or individuals who played games regularly or consistently in either a recreational (i.e., social gamers), competitive, or professional capacity (i.e., esports players). Studies that did not specifically recruit gamers were considered only if the data reported were stratified by game playing dose, exposure, duration, or frequency. The reasoning behind this decision was to discriminate regular or habitual gamers (as defined above) from a general population of non-gamers that would otherwise not self-identify as gamers or would not regularly engage with gaming. Gaming was defined as playing an electronic game on either a personal computer, game console (e.g., PlayStation, Xbox, Nintendo), or mobile phone. Studies involving arcade video game systems were therefore excluded. The search strategy was restricted to include studies published after January 2000 to examine only contemporary game-playing modalities.

Studies were included if they: (1) used an experimental trial design (e.g., randomized controlled trial, observational or prospective cohort study design), (2) reported data on habitual gamers, (3) used electronic gaming platforms, (4) had a sample of adult participants (i.e., aged 18 years or older); or in the case of studies with large samples with broad age ranges, had stratified the data by age (i.e., 18–30 years, 31–40 years, etc.), (5) reported at least one subjectively or objectively measured parameter of sleep, (6) were published in English and (7) were published from 2000 onward.

Studies were excluded if they used games as a stressor (i.e., to induce a physiological or psychosocial response), intervention tool (e.g., for educational or training purposes), or involved the use of gamified or simulated training or educational tools. The reasoning for this was to exclude studies that primarily examined the effects of game technologies on a general population group rather than habitually engaged gamers. Conference abstracts, other published abstracts, and gray literature (e.g., reports, white papers, academic theses, or dissertations) were also excluded.

### Study Selection

**FIGURE 1:**
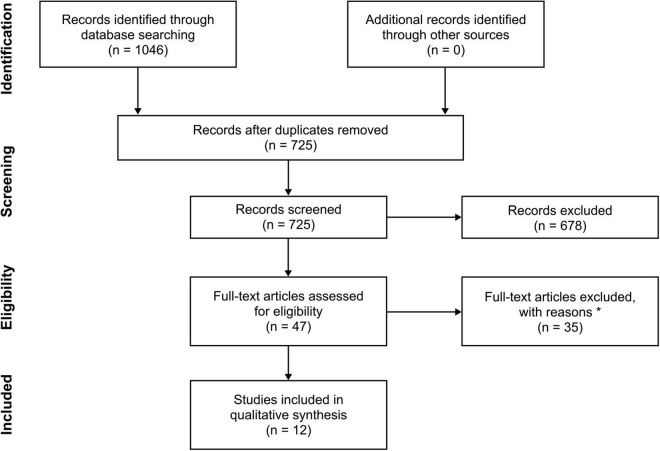
PRISMA diagram of study selection process. * No experimental group of habitual gamers (*n* = 19); no valid parameters of sleep or insomnia (*n* = 10); non-adult cohort (*n* = 4); unable to retrieve full text for appraisal (*n* = 1); article was retracted by the publishing journal (*n* = 1).

Figure 1 provides an outline of the study selection process. Studies were initially screened by assessing the title and abstract only. The appraisal of studies was performed by conducting full-text screening of the filtered studies using standardized methods by two independent reviewers (C.K. and D.T.R.). In the event of uncertainty or disagreement, disputes were resolved by consensus and arbitration (D.E.R.) or by contacting the corresponding authors.

## Results

### Synthesis of Evidence

The study selection process (Figure 1) yielded a total of 1046 records, of which 151 (14.4%) were extracted from PubMed; 317 (30.3%) from Scopus; and 578 (55.3%) from ISI Web of Science. The 321 duplicate records were removed, leaving 725 unique records eligible for screening. Of these, 47 records were identified for full-text appraisal, with the remaining 678 being excluded. A further 35 records were excluded during the appraisal process for the following reasons: (i) no experimental group of habitual gamers (*n* = 19); (ii) no valid parameters of sleep or insomnia were reported (*n* = 10); (iii) non-adult cohort was used (*n* = 4); (iv) unable to obtain the full-text and attempts to contact the authors had failed (*n* = 1); and (v) article was retracted by the publishing journal (*n* = 1). Therefore, 12 records were eligible for inclusion in the qualitative synthesis.

### Study Characteristics

The study characteristics are summarized in Table 2. Ten of the included twelve studies employed an observational study design (King and Delfabbro, 2009; Achab et al., 2011; Mario et al., 2014; Exelmans and Van den Bulck, 2015; Altintas et al., 2019; Evren et al., 2019; Akçay and Akçay, 2020; Ko et al., 2020; Rudolf et al., 2020; Wong et al., 2020), one study was a randomized crossover trial (Thomas et al., 2019) and another was a prospective cohort study (Thomée et al., 2015). All observational studies employed an online or paper-based survey approach, apart from two studies that also included face-to-face interviews (Exelmans and Van den Bulck, 2015; Ko et al., 2020), and one study that also performed clinical measurements in a controlled setting (Mario et al., 2014). Sample sizes of the included studies ranged from 9 to 1066 participants, with cohorts spanning over ten countries. Overall, the sex distribution of the studies was biased toward males and included: two studies with all-male populations (Mario et al., 2014; Thomas et al., 2019), six studies with populations that were majority male (range: 77 to 98% male) (King and Delfabbro, 2009; Achab et al., 2011; Thomée et al., 2015; Altintas et al., 2019; Ko et al., 2020; Rudolf et al., 2020) and four studies with populations that were majority female (range: 56 to 71%) (Exelmans and Van den Bulck, 2015; Evren et al., 2019; Akçay and Akçay, 2020; Wong et al., 2020). The cohorts used in six of the studies comprised gamers from the general population (King and Delfabbro, 2009; Achab et al., 2011; Exelmans and Van den Bulck, 2015; Thomée et al., 2015; Altintas et al., 2019; Rudolf et al., 2020) and one study cohort comprised an elite team of League of Legends esports players (Thomas et al., 2019). The rest of the included studies enrolled university students into their cohorts. The platforms that participants used to play games in each study varied between mobile, console, and computer gaming platforms. Four studies did not define the gaming platforms that participants used (Altintas et al., 2019; Evren et al., 2019; Ko et al., 2020; Wong et al., 2020), and three studies included participants who played games on the computer (Achab et al., 2011; Thomée et al., 2015; Thomas et al., 2019). The remaining studies did not discriminate between gamers by gaming platform and comprised cohorts using various gaming platforms. The studies poorly defined prior exposure to gaming. Only four studies reported the years of prior gaming exposure (range: less than 1.0 to 8.5 ± 6.7 years) (Achab et al., 2011; Mario et al., 2014; Akçay and Akçay, 2020; Ko et al., 2020). Three of these studies used gaming exposure as an inclusion criterion and subsequently did not report prior gaming exposure as an outcome measure (Mario et al., 2014; Akçay and Akçay, 2020; Ko et al., 2020). Gaming frequency was reported as hours spent playing games per day (range: 0.8 ± 1.0 to 10.3 ± 2.1 h) (Exelmans and Van den Bulck, 2015; Thomée et al., 2015; Thomas et al., 2019; Akçay and Akçay, 2020; Wong et al., 2020) or per week (range: less than 7.0 to 36.8 ± 22.0 h) (King and Delfabbro, 2009; Achab et al., 2011; Mario et al., 2014; Altintas et al., 2019; Ko et al., 2020; Rudolf et al., 2020). Only one study did not report gaming frequency (Evren et al., 2019).

**TABLE 2:** Demographic characteristics of the included studies.

Citation	Country	Study design	Population or subgroup	Age (years)	Sample size	%Male	Gaming platform	Gaming exposure	Gaming frequency
Achab et al. (2011)	France	DOS*%^a^*	Addicted (DAS +) and non-addicted (DAS-) MMORPG gamers	All: 26.6 ± 7.1 DAS+: 25.7 ± 6.5 DAS−: 27.0 ± 7.3	All: 448 DAS+: 123 DAS−: 325	All: 82.7 DAS+: 87.0*%^b^* DAS−: 81.2*%^b^*	PC	All: nr DAS+: 8.41 ± 5.93 y DAS−: 8.54 ± 6.66 y	All: 30.3 ± 18.7 h/wk DAS+: 36.8 ± 22.0 h/wk DAS−: 27.7 ± 16.7 h/wk
Akçay and Akçay (2020)	Turkey	DOS%^a^	University students	22.76 ± 2.21	892	29.5	PC, console, handheld console, mobile, tablet	Not playing to ≥5 y	Daily mean: 0.8 ± 1.0 h/day Weekday: 0.4 ± 0.9 h/day Weekend: 1.8 ± 1.6 h/day
Altintas et al. (2019)	France	DOS%^a^	Online game players with high (HSQ) and low (LSQ) sleep quality profiles	All: 24.40 ± 6.98 HSQ: 24.02 ± 6.77 LSQ: 24.64 ± 7.31	All: 217 HSQ: 132 LSQ: 85	All: 80.6 HSQ: nr LSQ: nr	nr	nr	All: 18.14 ± 17.90 h/wk HSQ: 17.52 ± 14.87 h/wk LSQ: 19.12 ± 21.85 h/wk
Evren et al. (2019)	Turkey	DOS%^a^	University students with (present) and without (absent) probable insomnia	All: nr Absent: 21.93 ± 3.50 Present: 21.51 ± 2.88	All: 1010 Absent: 810 Present: 200	All: nr Absent: 41.1 Present: 35.5	nr	nr	nr
Exelmans and Van den Bulck (2015)	Belgium	DOS	Adults in Flanders, Belgium	46.0 ± 17.76	844*%^c^*	43.8	PC, console, internet or social media games	nr	All: 22.8 min/day Gamers%^c^: 67.0 min/day
King and Delfabbro (2009)	Australia	DOS%^a^	Subgroup of heavy adult game players	20.1 ± 3.9	45	98.0	PC, console	nr	(i) >30.0 h/wk (ii) >4 days per week (iii) mean duration of 3 h/session
Ko et al. (2020)	Taiwan	DOS	Subgroups of university students who are regular gamers (RG) and IGD gamers (IGD)	RG: 24.59 ± 3.41 IGD: 25.32 ± 4.20	All: 138 RG: 69 IGD: 69	All: 78.3 RG: 78.3 IGD: 78.3	nr	> 2 y	RG:44.9% play >25 h/wk IGD: 97.1% play >25 h/wk
Mario et al. (2014)	United Kingdom	DOS	University students who are frequent (i.e., playing >7 h/wk) and infrequent (i.e., playing ≤7 h/wk) gamers	All: 21.0 (20.0 – 22.0) Frequent: 21.0 (20.0 – 22.0) Infrequent: 21.0 (21.0 – 22.0)	All: 45 Frequent: 21 Infrequent: 24	100	PC, console and exercise games	> 1 y	Frequent: >7 h/wk Infrequent: ≤7 h/wk
Rudolf et al. (2020)	Germany	DOS%^a^	Online gamers and esports players (including professional, former-professional, amateur, regular, and occasional players)	22.9 ± 5.9	1066	91.9	PC, console	nr	24.4 ± 15.9 h/wk *%^d^*
Thomas et al. (2019)	United States	RCT	Team of elite LoL esports players	20.8 ± 2.0	9	100	PC	nr	10.3 ± 2.1 h/day (LoL only) 1.8 ± 2.8 h/day (other games)
Thomée et al. (2015)	Sweden	PCS%^a^	A subgroup of male and female “high gamers” from a Swedish adult population registry who play games more than 2 h per day	Males: 21.9 ± 1.4 Females: 21.8 ± 1.3	Males: 319–325 Females: 96–99	∼76.8	PC, mobile, tablet%^e^	nr	Males: >2 h/day Females: >2 h/day
Wong et al. (2020)	China	DOS%^a^	University students	20.89 ± 1.48	300	40.67	nr	nr	1.12 ± 1.53 h/day

*Data are presented as mean ± standard deviation or median (interquartile range) unless otherwise indicated. DOS, Descriptive Observational Study; RCT, Randomized Crossover Trial; PCS, Prospective Cohort Study; MMORPG, Massive Multiplayer Online Role-Playing Game; PC, Personal Computer; IGD, Internet Gaming Disorder; GD, Gaming Disorder; LoL, League of Legends; nr, data not reported. ^a^ – exclusively survey-based; ^b^ – a calculated value representing the distribution of males per group, based on sample sizes reported in the study; ^c^ subset (34.4%) of the total population self-identified as gamers; ^d^ – a subset of total population (n = 978); ^e^ – mobile and tablet platforms were included in year five only.*

Five studies reported interactions between sleep and gaming addiction (Achab et al., 2011; Evren et al., 2019; Akçay and Akçay, 2020; Ko et al., 2020; Wong et al., 2020). A further four studies reported on interactions between sleep and gaming behavior (i.e., volume, duration, or intensity) (Mario et al., 2014; Exelmans and Van den Bulck, 2015; Altintas et al., 2019; Rudolf et al., 2020) and one study reported on interactions between sleep and physical health (Altintas et al., 2019). All of the studies employed self-report tools to measure parameters of sleep, which included the use of validated questionnaires such as the Pittsburgh Sleep Quality Index (PSQI; Buysse et al., 1989), Insomnia Severity Index (ISI; Bastien et al., 2001), Bergen Insomnia Scale (BIS; Pallesen et al., 2008), and Epworth Sleepiness Scale (ESS; Johns, 1991). Otherwise, studies also presented self-report data derived from non-validated means. Sleep characteristics reported across the included studies are presented in Table 3.

**TABLE 3:** Sleep characteristics reported by the included studies.

Citation	Self-reported Sleep duration (h)	PSQI (total score)	Other sleep parameters		
Achab et al. (2011)	All: 7.1 ± 1.3 DAS+: 6.8 ± 1.4 DAS−: 7.2 ± 1.3	nr	**Restful sleep (% No)**	**Sleep deprivation due to gaming (% Yes)**	**Daytime sleepiness (% Yes)**
	*p* = 0.043				
	OR 0.78, *p* = 0.004 ^b^		All: 19.1 DAS+: 53.5 DAS−: 46.5 *p* < 0.001 OR 0.23, *p* < 0.001 ^b^	All: 36.8 DAS+: 40.9 DAS−: 59.1 *p* < 0.001 OR 2.83, *p* < 0.001 ^b^	All: 22.7 DAS+: 48.0 DAS−: 52.0 *p* < 0.001 OR: 3.10, *p* < 0.001 ^b^

Akçay and Akçay (2020)	All: 7.5 ± 0.9 Stratified by gaming frequency:	All: 8.5 ± 2.7 Stratified by gaming frequency:	**ESS**	**Bedtime**	**Wake-up time**
	Not playing*^c^*: 7.2 ± 0.9	Not playing *^c^*: 8.2 ± 2.3			
	<2 h/day*^d^*: 7.6 ± 1.3 ≥2 h/day*^e^*: 7.5 ± 0.9 *p* < 0.001	<2 h/day^d^: 8.3 ± 2.7 ≥2 h/day^e^: 10.5 ± 3.0 *p* < 0.001	All: 3.0 ± 3.9 Stratified by gaming frequency: Not playing*^c^*: 4.0 ± 3.9 <2 h/day^d^: 2.6 ± 3.7 ≥2 h/day^e^: 4.9 ± 4.7 *p* < 0.001	All: 23.7 ± 1.8 Stratified by gaming frequency: Not playing^c^: 24.0 ± 0.9 <2 h/day^d^: 23.5 ± 1.9 ≥2 h/day^e^: 24.9 ± 1.8 *p* < 0.001	All: 7.4 ± 1.0 Stratified by gaming frequency: Not playing^c^: 7.3 ± 0.8 <2 h/day^d^: 7.3 ± 0.9 ≥2 h/day^e^: 8.5 ± 2.0 *p* < 0.001

Altintas et al. (2019)	nr	All: 6.24 ± 3.12 HSQ: 6.36 ± 2.99	**Good sleepers**	**Poor sleep**	
		LSQ: 6.07 + 3.32 *p* = 0.51		
			All: 44.70 HSQ: nr LSQ: nr	All: 55.30 HSQ: nr LSQ: nr	

Evren et al. (2019)	nr	nr	**ISI total score**		
	
			Absent: < 14 Present: > 14		

Exelmans and Van den Bulck (2015)	nr	4.56 ± 2.66 ^a^	**Bedtime (hh:mm)**	**Wake-up time (hh:mm)**	
	
			23:25 ± 1:05 ^a^	07:30 ± 1:25 ^a^	

King and Delfabbro (2009)	nr	nr	**SHI total score**		
			30.2 ± 7.1		

Ko et al. (2020)	nr	nr	**Time to fall asleep** **(<1am, 1–3am, >3am)**	**Wake-up time** **(<9am, 9am–12pm, >12pm)**	**Sleep duration** **(<4 h for > = 2 d/wk)**
	
			RG: 60.9%, 36.2%, 2.9 IGD: 17.4%, 52.2%, 30.4 *p* < 0.001	RG: 62.3%, 33.3%, 4.3 IGD: 30.4%, 37.7%, 31.9 *p* < 0.001	RG: 4.3 IGD: 58.0 *p* < 0.001

Mario et al. (2014)	nr	All: 6.0 (4.0 – 8.0) Frequent: 6.0 (5.0 – 9.0) Infrequent: 5.5 (4.0 – 7.0) *p* = 0.08			

Rudolf et al. (2020)	7.1 ± 1.3	nr	**Sleep quality [mode]**		
			“Quite good” (*n* = 642, 60.2%)		

Thomas et al. (2019)	8.1 ± 1.2	nr			

Thomée et al. (2015)	Males: 7.7 ± 1.1 Females: 7.9 ± 1.5	nr			

Wong et al. (2020)	nr	6.63 ± 2.14			

*Data are presented as mean ± standard deviation or median (interquartile range) unless otherwise indicated. BMI, body mass index; PSQI, Pittsburgh Sleep Quality Index; ESS, Epworth Sleepiness Scale; SHI, Sleep Hygiene Index; ISI, Insomnia Severity Index; nr, data not reported. ^a^ – subset (34.4%) of the total population self-identified as gamers; ^b^ – adjusted for age, sex and educational level; ^c^ – n = 195; ^d^ – n = 640; ^e^ – n = 57.*

Studies involving gaming addiction had calculated total scores using varying scales. However, they were all adapted or based on the Diagnostic and Statistical Manual of Mental Disorders (DSM; American Psychiatric Association, 2013) or International Classification of Diseases (ICD) criteria for substance dependence or internet game addiction. The scales that were used included: DSM-IV Substance Dependence Adapted Scale (DAS; Achab et al., 2011), Game Addiction Scale for Adolescents – Short Form (GAS-SF; Anli and Taş, 2018), Internet Gaming Disorder Scale – Short Form (IGDS9-SF) (Pontes and Griffiths, 2015), Young’s Internet Addiction Test – Short Form (YIAT-SF; Pawlikowski et al., 2013); and Addictive Intensity Evaluation Questionnaire (AIE-Q; Décamps et al., 2010).

### Sleep

Self-reported sleep duration was reported in five of the included studies, with gamers sleeping on average between 6.8 ± 1.4 to 8.1 ± 1.2 h per day (Achab et al., 2011; Thomée et al., 2015; Thomas et al., 2019; Akçay and Akçay, 2020; Rudolf et al., 2020). One study indicated that there was a greater occurrence of sleep duration under 4 h per night on at least 2 days per week among addicted gamers, compared to regular gamers (58.0% versus 4.3%, *p* < 0.001) (Ko et al., 2020). None of the studies that had reported on sleep specifically discriminated between nocturnal and diurnal sleep time. Sleep quality of gamers was reported subjectively in five studies using the PSQI (total scores range: 4.56 ± 2.66 to 10.5 ± 3.0) (Mario et al., 2014; Exelmans and Van den Bulck, 2015; Altintas et al., 2019; Akçay and Akçay, 2020; Wong et al., 2020). All studies reporting PSQI total scores, apart from Exelmans and Van den Bulck (2015) (PSQI total score: 4.56 ± 2.66), reported total scores greater than 5, thus indicating poor sleep quality.

### Sleep and Gaming Volume, Duration, and Intensity

Exelmans and Van den Bulck (2015) estimated gaming volume by (i) multiplying the volume of gaming during an average weekday (Mon – Thurs) by four; and weekend day (Sat and Sun) by two; (ii) summing the average measures of volume on weekdays, weekend days and Fridays; and finally (iii) dividing the result by seven to produce an average estimate game playing time (as hours per day). Weak, but significant, relationships between gaming volume with PSQI (*r* = 0.109, *p* < 0.01) and BIS (*r* = 0.104, *p* < 0.01) total scores; as well as bedtime (*r* = 0.172, *p* < 0.01), and rise time (*r* = 0.189, *p* < 0.01) were observed. Hierarchical cluster analysis further explained that an increasing volume of gaming was associated with worsened sleep quality (PSQI score: β = 0.145, *p* < 0.001), increased symptoms of insomnia (BIS score: β = 0.120, *p* < 0.01), as well as delayed bedtime (β = 0.100, *p* < 0.01) and rise times (β = 0.168, *p* < 0.001). Each additional hour of gaming per day delayed bedtime by 6.9 min [95% confidence interval (CI): 2.0–11.9 min], rise time by 13.8 min (95% CI: 7.8–19.7 min), and increased the odds of having poor sleep quality (defined as having a PSQI total score > 5) by 31.0% (95% CI: 1.09–1.57, *p* < 0.01). Compared to those who did not play games, gamers playing > 1 h per day had a greater chance of having poor sleep quality [Odds ratio (OR): 2.746, 95% CI: 1.596–4.723, *p* < 0.001], with 29.5% of the prevalence of poor sleep quality being attributed to gaming. Relatedly, these gamers were also more likely to have sleep latency (OR: 3.379, 95% CI: 1.699–6.720, *p* < 0.01) and sleep efficiency (OR: 2.776, 95% CI: 1.202–6.413, *p* < 0.01) PSQI sub-component scores of 2 (rather than 0) and were 2.29 times more likely to perceive their sleep quality as “rather bad,” than “very good,” compared to those who did not play games (*p* < 0.05). All models were adjusted for gender, age, educational level, hours of exercise, and perceived stress (1st step); and video gaming (2nd step).

Two studies did not find any association between gaming behavior and parameters of sleep (Mario et al., 2014; Rudolf et al., 2020). One of these studies calculated total video game playing (measured as hours per week) by summing the recorded hours of gaming over the last year in an average week (Mario et al., 2014). No significant association was found between total game playing and PSQI-measured sleep quality (rho = 0.23, *p* > 0.05) (Mario et al., 2014). The other study reported no associations between gaming duration (measured in hours per week) with self-reported sleep quality and duration (rho < 0.10, *p* > 0.05) (Rudolf et al., 2020).

### Sleep and Gaming Addiction

Achab et al. (2011) used the DAS to determine if gamers screened positive (DAS+) or negative (DAS−) for gaming addiction and were subsequently allocated to their respective groups. After adjusting for age, sex, and educational level, the DAS+ group were more likely to have shorter sleep duration (OR: 0.78, CI: 0.66–0.93, *p* = 0.004) and less restful sleep (OR: 0.23, 95% CI: 0.14–0.38, *p* < 0.001) compared to the DAS- group. There was also a greater frequency of sleep deprivation due to gaming (OR: 2.83, 95% CI: 1.83–4.38, *p* < 0.001) and daytime sleepiness (OR: 3.10, 95% CI: 1.92–5.00, *p* < 0.001) in the DAS+ group.

Altintas et al. (2019) did not find significant correlations between total PSQI scores and gaming variables (gaming duration: *r* = −0.035, *p* > 0.05; intensity of video game playing: *r* = −0.093, *p* > 0.05). Hierarchical cluster analysis was used to identify high sleep quality profiles (HSQ group; 60.83% of participants) and low sleep quality profiles (LSQ group; 39.17% of participants) amongst participants. After comparing PSQI subcomponent scores between these two groups, Altintas et al. (2019) showed that the LSQ group were characterized by having lower scores of subjective sleep quality (2.84 ± 0.75 versus 1.66 ± 0.56, *p* = 0.01) and sleep efficiency (0.51 ± 0.84 versus 0.11 ± 0.33, *p* = 0.01); greater scores of sleep latency (2.04 ± 0.73 versus 0.60 ± 0.55, *p* = 0.01), sleep disturbance (1.25 ± 0.55 versus 0.78 ± 0.53, *p* = 0.01) and sleep medication use (0.37 ± 0.88 versus 0.03 ± 0.27, *p* = 0.01). The LSQ group also had greater video game duration (19.12 ± 21.85 h/week versus 17.52 ± 14.87 h/week, *p* = 0.01) and intensity of video game playing total scores (44.33 ± 16.86 versus 37.21 ± 11.51, *p* = 0.01), compared to the HSQ group. Furthermore, in logistic regression analysis, high intensity of video game playing was associated with a greater risk of having low sleep quality (OR: 0.969, 95% CI: 0.946–0.993, *p* = 0.01) when sleep quality profile was used as the dependent variable.

A recent study by Akçay and Akçay (2020) reported positive correlations between GAS-SF scores and various self-reported or subjective parameters of sleep, including daily average sleep duration (measured in hours; *r* = 0.118, *p* < 0.001), wake-up time (*r* = 0.114, *p* = 0.001) and total scores for PSQI (*r* = 0.247, *p* < 0.001) and ESS (*r* = 0.066, *p* = 0.048). More extended periods of gaming (β = 0.108, *p* < 0.001) and the presence of technology in the bedroom (β = 0.203, *p* < 0.001) were also associated with poorer PSQI-measured sleep quality. Together, these two factors independently contributed to 38% of the variance in the study, after controlling for age, sex, family income, tertiary education year, place of residence, consumption of caffeinated drinks, alcohol, substance abuse, and smoking. Similarly, participants who indicated the presence of a technological device in the bedroom also had lower average sleep duration (7.4 ± 1.1 h versus 7.6 ± 0.9 h, *p* = 0.015) and higher mean GAS-SF (21.1 ± 10.5 versus 15.4 ± 7.6, *p* < 0.001), total scores for PSQI (10.3 ± 3.0 versus 8.0 ± 2.4, *p* < 0.001) and ESS (4.9 ± 4.3 versus 2.6 ± 3.6, *p* < 0.001), compared to those who did not.

Evren et al. (2019) used the ISI to discriminate presence or absence of probable insomnia (using the ISI total score > 14) among students engaged with esports or gaming. Stepwise linear regression analysis demonstrated positive associations (β = 0.107, *p* < 0.001) between insomnia and internet addiction severity (measured with the YIAT-SF) after adjusting for anxiety, depression, neuroticism, and dimensions of attention deficit hyperactivity disorder (inattentiveness; hyperactivity or impulsivity). Additionally, the group with a presence of probable insomnia was characterized by greater YIAT-SF internet addiction severity total scores, compared to the group without insomnia (31.71 ± 9.38 versus 26.48 ± 8.23, *p* < 0.001).

Wong et al. (2020) explored the relationship between IGD severity with sleep quality and emotional distress in young adults and reported positive correlations between IGDS9-SF and PSQI total scores (*r* = 0.249, *p* < 0.001). Furthermore, this relationship was independently maintained (β = 0.157, *p* < 0.05) even after adjusting for age, gender, time spent on smartphones, time spent on social media, and time spent gaming in multiple regression analyses.

Ko et al. (2020) investigated delayed sleep phase syndrome (DSPS) and insomnia in gamers and healthy controls. DSPS and Insomnia Disorder were confirmed based on standardized criteria during face-to-face interviews with an experienced psychiatrist specializing in internet gaming disorder. Chi-squared analysis revealed that there was a greater occurrence of DSPS (43.5% versus 2.9%, *p* < 0.001) and insomnia disorder (17.4% versus 4.3%, *p* < 0.05) among gamers with internet gaming addiction (IGD group; classified by DSM-V criteria), compared to healthy controls. These results were mirrored in gamers with gaming disorder (GD group; classified by ICD-11 criteria) who also had a greater occurrence of DSPS (36.4% versus 2.9%, *p* < 0.001) and insomnia disorder (22.7% versus 4.3%, *p* < 0.01) when compared to healthy controls.

### Sleep and Physical Health

Altintas et al. (2019) also assessed physical health and was the only study to report associations between sleep quality and physical health. Sleep quality was assessed using PSQI total scores, and physical health was assessed with the 36-item Short Form (SF-36) generic health survey (Ware and Sherbourne, 1992). No significant relationship was found between PSQI total scores and physical health (*r* = 0.049, *p* > 0.05). Likewise, no relationships were found using logistic regression analyses with either high or low sleep quality profiles and physical health (OR: 1.005, 95% CI: 0.964–1.048, *p* = 0.81).

### Gaming Behavior and Cardiometabolic Disease Risk

This section characterizes outcome measures that do not align with the primary narrative but provide insight into gamers’ current health and lifestyle behaviors.

BMI was reported in six of the included studies (King and Delfabbro, 2009; Mario et al., 2014; Thomée et al., 2015; Thomas et al., 2019; Rudolf et al., 2020; Wong et al., 2020). Only two of these studies objectively measured anthropometric outcomes (Mario et al., 2014; Thomas et al., 2019); the rest of the studies relied on self-reported data. BMI ranged from 20.52 ± 2.64 kg.m^–2^ (Wong et al., 2020) to 25.60 ± 3.44 kg.m^–2^ (Thomas et al., 2019). The prevalence of being overweight among gamers was reported to be as high as 31.0 and 38.0% (King and Delfabbro, 2009; Thomée et al., 2015). In a different study, only 51.3% of the population was classified as normal weight (Rudolf et al., 2020).

One study reported that frequent gamers (i.e., playing > 7 h per week) had greater waist circumferences (median: 84.5, IQR: 77.7–101.3 cm, *p* = 0.04), fat masses (median: 12.3, IQR: 7.2–19.9 kg, *p* = 0.04) and heart rates (median: 85.6, IQR: 69.0–97.3 bpm, *p* = 0.007) compared to infrequent gamers (median: 81.8, IQR: 77.6–86.5 cm; median: 9.5, IQR: 8.0–13.0 kg; median: 76.5, IQR: 63.0–80.6 bpm, respectively) (Mario et al., 2014). No differences were observed for body weight, height, systolic or diastolic blood pressure between these groups (Mario et al., 2014). Similarly, another study also found no differences in BMI between professional, former professional, amateur regular, and occasional gamers (Rudolf et al., 2020). Compared to a prospective cohort study population at baseline, however, it was indicated that the mean BMI of both male (24.2 ± 4.1 kg.m^–2^, *p* < 0.05) and female (24.1 ± 4.9 kg.m^–2^, *p* < 0.01) “high gamers” (i.e., playing games > 2 h per day) were significantly greater than the male and female “low gamers,” respectively (Thomée et al., 2015).

Three studies described associations between gaming behavior with health and physical activity (Mario et al., 2014; Thomée et al., 2015; Rudolf et al., 2020). Mario et al. (2014) reported that total video game playing time (h/wk) correlated positively with body mass index (BMI; rho = 0.30, *p* < 0.05), waist circumference (rho = 0.42, *p* < 0.01), fat mass (rho = 0.47, *p* < 0.01) and heart rate (rho = 0.47, *p* < 0.01). Total vigorous physical activity (measured as metabolic equivalent minutes per week; MET min/week) was negatively correlated with total video game playing time (rho = −0.30, *p* < 0.05).

Rudolf et al. (2020) reported similar (albeit weaker) positive correlations between video gameplay time (h/wk) with BMI (rho = 0.11, *p* < 0.01) and sedentary behavior (rho = 0.15, *p* < 0.01) after adjusting for age, gender, and education. Additionally, the group observed a negative association between video gameplay time and self-reported health status (rho = −0.12, *p* < 0.01) after adjusting for BMI and sedentary behavior.

Thomée et al. (2015) assessed associations between leisure computer use among “high gamers” (i.e., playing > 2 h per day) and prevalence of overweight (i.e., BMI ≥ 25 kg.m^–2^) at baseline and follow-up, 1–5 years later. At baseline, the prevalence of overweight was 38% and 32% in males and females, respectively. In cross-sectional logistic analyses adjusting for age, occupation, physical activity, social support, and sleep duration, the prevalence of overweight at baseline was greater in the “high gamers” males (OR: 1.70, 95% CI: 1.30–2.26, *p*-value not reported) and females (OR: 1.70, 95% CI: 1.06–2.74, *p*-value not reported), compared to “low gamers.” Similarly, the prevalence of obesity (BMI ≥ 30 kg.m^–2^) at baseline was higher in the “high gamer” males (OR: 1.8, 95% CI: 1.02–3.04) and females (OR: 2.1; 95% CI: 1.02–4.47) compared to the “low gamers.” For prospective analyses, participants with baseline BMI ≥ 25 kg.m^–2^ were excluded. In adjusted models, at baseline, female “high gamers” were more likely to be overweight at 1-year follow-up (OR: 3.2, 95% CI: 1.23–8.12). This was associated with new cases of being overweight at 5-year follow-up (OR: 3.0, 95% CI:1.29–6.83) after adjusting for occupation and physical activity. Accounting for BMI changes, this translated to a total increase in BMI from baseline to 5-year follow-up of 2.12 BMI units in female “high gamers.” These associations were not seen amongst male “high gamers.”

Outcomes of health and physical activity were reported in six of the included studies (King and Delfabbro, 2009; Mario et al., 2014; Thomée et al., 2015; Thomas et al., 2019; Ko et al., 2020; Rudolf et al., 2020). Ko et al. (2020) reported a higher frequency of poor health and lifestyle behaviors among addicted gamers (IGD group) compared to those with no gaming addiction (CON group). This was characterized by a greater occurrence of overweight/obesity (defined as BMI > 27 kg.m^–2^; 27.5% versus 14.5%, *p* < 0.01), irregular diet (13% versus 2.9%, *p* < 0.05), increased body weight (11.6% versus 1.4%, *p* < 0.05), and unhealthy behavior (defined broadly as problems resulting from online gaming engagement; 95.7% versus 15.9%, *p* < 0.001) in the IGD compared to CON group. More gamers in the IGD group (40.6%) reported no exercise for more than 3 weeks compared to the CON group (0%, *p* < 0.001), and more gamers in the IGD group (73.9%) reported being sedentary for more than 4 h on three or more days per week, compared to the CON group (2.9%, *p* < 0.001).

Similar results were reported in other studies involving frequent or high-volume gamers (Mario et al., 2014; Thomée et al., 2015). In one study (Mario et al., 2014), frequent gamers (i.e., playing > 7 h per week) were found to engage in less total vigorous physical activity (MET min/wk) compared to infrequent gamers (median: 0, IQR: 0–3800 versus median: 960; IQR: 0–3720, *p* = 0.04). Another study reported that the subgroups of male and female “high gamers” (i.e., playing more than 2 h per day) reported less time spent doing regular or vigorous physical activity (males: 33% versus 49%, females: 17% versus 41%, *p* < 0.001) activity and more time being physically inactive (males: 34% versus 17%, females: 40% versus 13%, *p* < 0.001), compared to the entire study group (Thomée et al., 2015).

Interestingly, most gamers reported themselves to be in either good (38.6%), very good (38.2%), or excellent (18.2%) health (Rudolf et al., 2020). In this study, only a small group of gamers reported having poor (4.8%) or very poor (0.2%) health status. More than two-thirds (66.9%) of gamers in this study reported engaging in moderate to vigorous physical activity lasting more than 2.5 h per week and reported mean sedentary behavior time was 7.7 ± 3.6 h per day across the entire cohort. There were no significant differences in sedentary behavior time between the different types of gamers (*p* = 0.34). The sporting activities that these gamers reported engaging in were diverse, ranging from yoga and Pilates (1.9%) to jogging (28.3%) and fitness training (36.0%). Only 16.5% of gamers reported not participating in any sporting activity at all. The study also reported no statistically significant or relevant (rho <0.10) associations between video gameplay time and physical activity.

Similarly, in a separate study using heavy gamers [defined as those “(a) playing for over 30 h per week, (b) playing for at least 4 days per week, and (c) playing for an average duration of 3 h in a typical sitting”] one in five (20%) reported exercising for at least 30 min on three or 4 days per week, while one in four (24%) exercised less than once per week (for 30 min), and nearly one in five (18%) did not exercise at all (King and Delfabbro, 2009). In comparison, elite esports players reported exercising at a frequency of 4.2 ± 1.7 days per week (Thomas et al., 2019).

## Discussion

Based on the reviewed evidence, it appears that excessive gaming activity is broadly associated with worsened parameters of sleep. Gamers with high gaming addiction scores were vulnerable to poor sleep and were more likely to have shorter sleep duration, poorer quality sleep, and greater daytime sleepiness and insomnia scores than gamers with low gaming addiction scores and non-gamers. These findings were mirrored in gamers with high gaming volume and duration, who were more likely to have worsened sleep quantity and quality, in addition to delayed sleep timing and a greater prevalence of insomnia. Therefore, it is apparent that sleep problems may not be limited to addicted gamers; but could be the culmination of various factors related to gaming exposure in general.

Exelmans and Van den Bulck (2015) indicated that decrements in sleep quality became most profound after gaming exceeded a volume of 1 h per day. Akçay and Akçay (2020) corroborated these findings, demonstrating unfavorable effects on sleep resulting from gaming for more than 2 h per day. This was in line with a previous study conducted in adolescents, which showed that playing games for 50 min per day resulted in nearly no disruption on initiation or maintenance of sleep, with no changes to sleep structure (Weaver et al., 2010). Consequently, these results point toward a lack of consensus regarding the dose at which gaming becomes problematic. Moreover, these results highlight the heterogeneity in these studies since it may not be possible to arrive at the same dose-dependent association regarding the effect of gaming on sleep in both adults and adolescents, whose reward networks are still maturing (Reichelt, 2016).

It is also speculative whether sleep decrements attributed to gaming exposure exhibit a cumulative (rather than an acute) effect or whether other factors related to gaming behavior are involved. Altintas et al. proposed the concept of gaming intensity, highlighting that it may be a more salient factor as a predictor of sleep quality than gaming duration (Altintas et al., 2019). In traditional sports, athletes engaged with high-intensity sports were characterized with significant differences in sleep patterns (including better sleep structure and sleep continuity) compared to those engaged in low-intensity sports (Suppiah et al., 2015). It is, therefore, arguable whether this extends to gamers engaged with high-intensity gaming, such as esports athletes. Indeed, these gamers may not match the levels of physical exertion experienced by traditional athletes; however, the level of intensity may be comparable to the cognitive effort (or “mental fitness”) required to maintain their proficiency and competitive status (Kemp et al., 2020). While this is speculative, it is likely that novel performance metrics, such as actions per minute (APM), which measure game input commands, could be used as an objective proxy for gaming intensity in the future (Bonnar et al., 2019). Of course, the intensity of their lifestyle and rigorous training schedules could also explain sleep issues, which is apparent from the high rates of burnout and fatigue reported amongst these gamers (ESPN, 2015).

Realistically, the mechanisms driving the adverse effects on sleep may involve the coupled interaction of behavioral and physiological factors related to gaming activity. Most notable is the inhibitory effect that short, blue wavelength light emitted from device screens has on melatonin. The suppression of nocturnal melatonin secretion from the pineal gland delays the sensation of sleepiness and thus the onset of sleep (Gooley et al., 2011). This effectively reduces sleep quality by increasing sleep onset latency time. Sleep structure is also impaired because of nighttime light exposure, thereby further impairing the quality of sleep (Blume et al., 2019). It is, however, speculative whether the efficacy of these interactions is more or less profound during daytime gaming, which questions the extent that timing (rather than duration) of gaming sessions contributes toward problematic sleep. Likewise, light sources (from screen devices) attached to gaming modalities may also presumably exhibit varying effects on the circadian clock and thus sleep, depending on the inclusion of light sources that are most stimulatory for photoentrainment.

Similarly, gamers exposed to light sources emitting greater intensity light, or gamers situated near such light sources, may presumably experience more remarkable chronobiological effects and thus be at greater risk for problematic sleep. This could be explained by artificial light sources having variable spectral profiles in terms of the distribution of short- and long wavelength light (Blume et al., 2019). The size or configuration of screen devices may also affect light fluence received by gamers (Lei et al., 2013). These factors have not been explored and warrant further investigation.

Physiological arousal resulting from gaming activity may also be a factor and has previously been proposed as an underlying mechanism involved in difficulty initiating and maintaining sleep (Exelmans and Van den Bulck, 2015). Indices of physiological arousal that may be negatively affected by gaming activity include respiratory rate, blood pressure, and heart rate (Exelmans and Van den Bulck, 2015). Furthermore, since gaming intensity is complementary to gaming duration in its deleterious effect on sleep (Altintas et al., 2019), it is possible that certain games (e.g., those which could be considered more intense or stimulating) would exhibit stronger effects on arousal (and thus sleep), compared to other games. For instance, games with high actions per minute may be faster-paced and thus induce greater alertness and require greater attention. Therefore, cognitive alertness may also be a key factor impairing efforts to initiate sleep. This effect was demonstrated by Mathiak and Weber (2006), who used functional magnetic resonance imaging to show heightened cognitive alertness in a cohort of experienced gamers playing a first-person shooter game.

Interestingly, sleep was not associated with cardiometabolic health in the single study that considered it. Indeed, a robust relationship may exist between sleep and cardiometabolic disease risk in gamers, although the relatively young age of gamers may also mask this (i.e., since cardiometabolic diseases typically present with aging). The few studies that reported health parameters, independent of their sleep, characterized gamers as having poorer health and more unhealthy lifestyle behaviors. Among these unhealthy behaviors were reported lower levels of physical activity and higher levels of sedentary behavior, although the dose, frequency, and intensity of physical activity varied across the included studies. Gaming duration was also positively associated with BMI, waist circumference, fat mass, and heart rate. Addicted and “heavy” (or frequent) gamers appeared to be the most sedentary relative to other groups of gamers, which would be expected given their high volume of gaming activity. Conversely, most esports players met the WHO’s global recommendations for physical activity (World Health Organization, 2010), despite reporting as much as 24.4 ± 15.9 h of gaming per week (Rudolf et al., 2020).

Sedentary behavior is recognized as an independent determinant of poor health and has previously been associated with insomnia and sleep disturbances (Yang et al., 2017; Martínez-Ramos et al., 2018). Prolonged sitting time from gaming could therefore explain the adverse health outcomes reported. Coupled with poor sleep, sedentarism and poor lifestyle behaviors may only act to exacerbate the long-term cardiometabolic risk in an already vulnerable population. This is explained by a population where as much as 38% of gamers were overweight (Thomée et al., 2015). Moreover, particular groups of gamers, including addicted gamers, heavy or high-volume gamers, and female high-volume gamers, were identified as vulnerable risk groups for adverse health outcomes. Therefore, targeted interventions such as educational awareness, health prevention, and behavior change programs would be beneficial and necessary in mitigating associated health deficits in these cohorts. Finally, none of the included studies reported any associations between sleep and gaming performance, indicating a major literature gap. We echo that, indeed, the performance of gamers and esports athletes may be vulnerable to the adverse effects of short and poor-quality sleep (Bonnar et al., 2019). Therefore, future work should seek to identify the factors involved and establish informed, evidence-based recommendations or programs to support the performance management of these gamers (Bonnar et al., 2019).

### Limitations of Reviewed Studies

Despite the suggestive nature of these findings, it is essential to acknowledge and address the limitations related to the design of the included studies. The first major limitation present across nearly all studies involved the use of self-report instruments (not including validated subjective tools like the PSQI) to measure sleep parameters. Consequently, the findings presented in these studies are subject to several potential issues, including both social desirability and recall biases. This is particularly the case for self-reported sleep duration, where there is a rich history of over-reporting (Girschik et al., 2012). Considering that sleep was seldom the primary outcome of interest in the reviewed studies, it is sensible that a low-cost, practical alternative was employed over objective measures, such as actigraphy or polysomnography. However, the use of self-report instruments is discouraged since it may reduce the ability to identify significant causal or associative relationships between sleep with gaming behavior and health. Likewise, this issue extrapolates to self-reported gaming behaviors (particularly gaming duration and exposure) as well. On the one hand, gamers may inadvertently over-report their daily playing time, failing to discriminate between active engagement and idle gaming time (e.g., in lobbies, matchmaking queues, and during pause time). On the other hand, gamers may deliberately over-report or under-report their gaming time for social favor or to protect the public image of gaming, respectively.

The second limitation was the use of cross-sectional study designs, which do not allow for causal inferences. The majority of observational studies employed the sole use of online or paper-based survey data collection methods, which further reduces the integrity of the reported data for reasons previously discussed. Additionally, data collection methods solely using online surveys could be subject to deliberate and fraudulent false reporting owing to the anonymity of the online surveys used. A recent study highlighted a growing issue where respondents may intentionally submit fraudulent data by submitting duplicate, inconsistent or low-quality responses (Pozzar et al., 2020). The same research group also indicated sophisticated ways in which “fraudsters” could distort data by using robotic automation (or “botting”), virtual private networks, and server farms to generate mass fraudulent responses (Pozzar et al., 2020). While the occurrence of fraudulent data may be rare, researchers should be mindful of such threats to the validity of their sample and, thus, the integrity of their data.

The third limitation included the use of convenience samples and sampling methods that reduce the generalizability and representativeness of the participants. Researchers should use random sampling methods to ensure that their study populations are broadly representative and transferable to other age groups, sexes, and nationalities. This may also reduce the risk for social desirability and sampling biases, especially when sampling individuals in non-clinical settings. Relatedly, the relatively small share of female gamers among the considered study populations of the reviewed studies is of particular concern. This methodological oversight creates a reporting bias that limits the applicability of evidence to clinical recommendations for both sexes. Beyond this, it also perpetuates the false narrative that females comprise a minority of the general gaming cohort, where in fact, they comprise nearly half of the general gaming demographic (Entertainment Software Association, 2019).

A fourth limitation concerns the variability in the outcomes related to sleep reported in each of the studies. Only PSQI and sleep duration were consistently reported. Therefore, miscellaneous sleep metrics should be interpreted with caution. This is particularly the case for metrics that were not based on validated questionnaires.

The fifth limitation regards the self-reported sleep duration. Based on the reviewed evidence, gamers would appear to meet the recommended guidelines for sleep duration between 7 and 9 h for adults, as outlined by the National Sleep Foundation (Hirshkowitz et al., 2015). While these findings do not appear clinically significant, it is essential to note that these data do not discriminate between (and may thus represent a composite of) diurnal and nocturnal sleep time. Therefore, the interpretation of these findings is cautioned since the purported sleep duration may not reflect nocturnal sleep.

The sixth limitation regards the lack of results concerning circadian disruption. Again, the methodological oversight by studies not employing objective tools, such as actigraphy, arguably restricts researchers’ ability to develop a holistic understanding, particularly regarding the respective roles of other contributing factors, on problematic sleep and the potential downstream effects on health and performance. In particular, the pervasive phase-delaying and melatonin-suppressing effect of light from gaming at nighttime may adversely affect circadian rhythms, the sleep-wake cycle, and sleep (Ceranoglu, 2014; Blume et al., 2019); and cannot be quantified by subjective means.

Lastly, studies did not discriminate between the different types of gamers and often assumed homogeneity across their gaming populations. This major limitation is discussed in more depth in the following section, which aims to provide direction to researchers involved in future research.

### Implications for Policy and Practice

Gaming addiction tests appear to be criterion methods to discriminate between pathological and non-pathological gaming behavior, but this may be confounded by competitive gamers (such as esports athletes) who may engage in prolonged periods of online gaming without functional impairment. Therefore, researchers should apply the appropriate tools to their respective study populations to limit bias and erroneous reporting. Beyond this, it is apparent that there is a great deal of heterogeneity in the instruments used to assess gaming and internet addiction, which provoked the call for the validation of standardized procedures toward the identification and management of these gamers (Costa and Kuss, 2019). Achab et al. (2011) highlighted that there is also a need for validated tools in determining internet and genre-specific gaming addiction, arguing that previous studies do not differentiate between internet use in general (e.g., browsing, social media, and other general internet-use activities) and online games, nor between different types of online games. Another research group concluded that addicted and non-addicted gamers demonstrated varying physiological arousal deficits, depending on the game genre (Metcalf and Pammer, 2013). Ultimately, appropriating effective research methodology may improve researchers’ understanding regarding the etiology of gaming addiction and comorbid sleep problems, which may assist with the early discovery of pathological gaming habits, education around “healthy” gaming practices, interventions, and other related treatment strategies.

Researchers should also endeavor to describe their gaming cohorts more coherently. In the case of studies using heterogeneous gaming cohorts, researchers should consider prior years of gaming exposure; current frequency, duration, and intensity of gameplay; in addition to the current level of competitive involvement (i.e., social, amateur, novice, semi-professional, or professional) of players as potential factors that may affect outcomes of interest. For example, esports athletes may have greater overall skill and exposure to gaming than social gamers. Esports athletes may also embark on frequent transmeridian travel or engage in online events scheduled for a different time zone to where they live, which may invariably confound outcomes related to sleep and circadian rhythms. Moreover, these gamers may also have little to no restrictions on their playtime and may not curtail sleep in favor of gaming practices or matches, compared to non-professional gamers who have (in addition to their gaming) other lifestyle commitments, such as work or studies. Therefore, a cohort comprising both social gamers and esports athletes may yield dampened significance or confounded findings.

By extension, researchers are also cautioned against including heterogeneous cohorts comprising gamers playing on disparate gaming platforms (i.e., computer, console, or mobile). Although gamers playing on a single modality may represent a proportionately small group of gamers, it is unknown how individual differences between each gaming platform impact sleep, circadian rhythms, and other related physiological outcomes. For example, gamers playing on computers, consoles, mobile, or even virtual reality devices may experience varying chronobiological effects, based on their (i) proximity to screen devices, (ii) viewing geometry, and (iii) dose and intensity of light exposure from screens. Thus, the corresponding effects on sleep patterns may also differ. In addition, gameplay time may also be differently affected; for example, mobile gamers may be limited in gaming time by the battery span of their mobile devices, while PC and console gamers are not. Likewise, virtual reality gaming, in addition to increased light exposure from the headset, may offer greater immersion with the game than other gaming modalities, which may augment physiological arousal and thus, contribute to worsened sleep.

It is clear from the synthesis of evidence that further longitudinal research is warranted to establish causal inferences with gaming on sleep, cardiometabolic health, and performance. Additionally, longitudinal studies may be more effective in informing the direction of developmental trends regarding gaming behaviors and problematic sleep. Research may also benefit from describing gamers’ sleep in more detail, including using multiple validated questionnaires combined with objective sleep monitoring. Employing objective sleep monitoring, such as actigraphy or polysomnography, and using clinical health measures may benefit researchers by reducing the intrinsic biases associated with subjective data collection methodology. This may assist in establishing a better empirically grounded understanding of the mechanics driving problematic sleep through gaming behavior, which is currently lacking because of the heterogeneity in reporting of sleep parameters by the included studies. Likewise, future research is encouraged to use sleep diaries through which study participants may detail their sleep, daily gaming activities, caffeine consumption, physical activity, medication, and supplement use; to reduce recall bias and to provide a greater level of detail regarding participants’ free-living behaviors and the factors influencing their sleep. Finally, researchers should seek a more representative and generalizable cohort of gamers, with a more accurate share of female gamers to improve the application of evidence toward treatment strategies, future studies, and policymaking. The present body of evidence lacks sufficient credence to warrant the establishment of policies or recommendations for esports practice and regular engagement with gaming. Instead, researchers are encouraged to appropriate the suggestions above and develop training strategies that include sleep hygiene education and awareness and incorporate exercise or related techniques to improve sleep. Incorporating cognitive behavioral therapy for insomnia framework has also been recommended to prevent and treat sleep disturbances in esports players (Bonnar et al., 2019).

### Limitations of the Systematic Review

Beyond the limitations previously discussed, we also acknowledge the limitations of the present systematic review. The first limitation was the sole inclusion of studies published in the English language. It is possible that related studies, which may have been published elsewhere in other languages, may not have been accounted for. This is particularly the case since gaming and esports have both been globally received in recent years. The second limitation is that the search strategy employed in the present review may not include all relevant studies related to gaming and esports, especially in cases where they have been described differently. This follows the condition that there is a lack of consensus regarding esports’ proper definition and spelling. While attempts were made to use globally inclusive search terms previously used to describe esports (such as “cybersports”), other terms may have been missed. Lastly, the present review only included studies involving adults. There is a plethora of research on gaming involving children and adolescents; therefore, these findings may be limited to only adult gaming cohorts.

## Conclusion

We corroborate the findings reported in previous systematic reviews that excessive gaming is associated with worsened sleep parameters. We contribute to the existing literature by providing evidence related to adults habitually engaging with gaming (rather than children and adolescents) and offer direction for future work in this field. We also draw attention to esports and encourage researchers to explore this rapidly growing phenomenon. In addition, we encourage researchers to explore further the coupled interaction involving poor quality and quantity sleep with cardiometabolic health and gaming performance in adult gamers, as well as the relative contribution (and associated risk) that various games, game genres, and timing of the gaming activities have on sleep. Finally, we note that the growing access to technology and media (particularly in the bedroom at nighttime), and not only gaming, appears to have a massive impact on the development of problematic sleep among young adults. Therefore, researchers are encouraged to explore interventions that may attenuate related deficits, particularly in vulnerable cohorts, such as high volume and addicted gamers.

## Data Availability Statement

The original contributions presented in the study are included in the article/supplementary material, further inquiries can be directed to the corresponding author.

## Author Contributions

CK and DER: conceptualization and methodology. CK: formal analysis and writing—original draft preparation. CK and DTR: filtering and processing. CK, PP, DTR, DER, LR, and GL: writing—review and editing. DER, LR, GL, and PP: supervision. All authors contributed to the article and approved the submitted version.

## Conflict of Interest

The authors declare that the research was conducted in the absence of any commercial or financial relationships that could be construed as a potential conflict of interest.

## Publisher’s Note

All claims expressed in this article are solely those of the authors and do not necessarily represent those of their affiliated organizations, or those of the publisher, the editors and the reviewers. Any product that may be evaluated in this article, or claim that may be made by its manufacturer, is not guaranteed or endorsed by the publisher.
